# How Can Diet Influence the Risk of Stroke?

**DOI:** 10.1155/2012/763507

**Published:** 2012-05-30

**Authors:** Fernanda Medeiros, Marcela de Abreu Casanova, Julio Cesar Fraulob, Michelle Trindade

**Affiliations:** ^1^Department of Applied Nutrition, Federal University of the State of Rio de Janeiro, 20270004 Rio de Janeiro, RJ, Brazil; ^2^Department of Clinical Medicine, State University of Rio de Janeiro, 20551-030 Rio de Janeiro, RJ, Brazil

## Abstract

Cerebrovascular diseases are the second cause of mortality in the world, and hypertension is considered a main risk factor for occurrence of stroke. The mechanisms responsible for the increased stroke risk remain unclear. However, dietary interventions have been applied in the management and treatment of their risk factors, which include increased blood pressure levels, obesity, diabetes, and dyslipidemia. Further studies should be conducted to assess the effects of carotenoids, flavonoids, n-3 polyunsaturated fats, and lower salt and high glycemic index intake in risk of stroke.

## 1. Introduction

The main risk factor for stroke is high blood pressure (BP) and, when properly controlled, significantly reduces the incidence rates of this disease. Despite all advances achieved in recent years, its prevention is a priority, and in this respect, BP control has a prominent role [1]. An increase has been observed in the number of cerebrovascular events in developing countries that matches with food and lifestyle changes arising from industrialization and urbanization [2, 3]. Accurately assessing and understanding the role of nutrition in the causes and consequences of stroke will be crucial in developing and implementing strategies to minimize the global burden of stroke [4]. The aim of this paper is to describe the evidence linking nutrition to the risk of stroke.

## 2. Role of Nutrients for Stroke Prevention

### 2.1. Salt

Salt is an essential substance to man and to all types of animal life. We can see the important role of salt through the records of human history. However, during the past century, the evidence for the risks imposed on human health by excess salt consumption has become compelling. The causal relationship between habitual dietary salt intake and BP has been established through experimental, epidemiological, and even intervention studies. Most adult populations around the world have average daily salt intake higher than 6 g per day although international recommendations suggest that average population salt intake should be less than 5-6 g per day [4–6].

The relationship between salt intake and health is recognized by the scientific community. Initially, this relationship was made with the association between magnitude of salt intake and hypertension. Two achievements make a major impact on this subject, the quantitative measurement of dietary sodium intake and repeated recognition that the greater the daily sodium intake, the higher the prevalence of hypertension in populations [7]. A series of population-based intervention studies and randomized controlled clinical trials have shown that it is possible to achieve significant reductions in BP with reduced salt intake in people with and without hypertension [8–12].

The Intersalt multicenter study that included normotensive and hypertensive patients was conducted in 52 centers from different countries in the 80s. This study showed that dietary intake of sodium (100 mmol/day) was associated with differences in systolic pressure of approximately 2.2 mmHg after adjustment for age, gender, excretion of potassium, body mass index, and alcohol intake [10].

Meta-analysis of intervention studies with salt restriction showed reductions in systolic BP (3.7 to 7.0 mmHg) and in diastolic BP (0.9 to 2.5 mmHg) in hypertensive patients [8, 13]. The response of BP to sodium reduction is direct and progressive, but nonlinear; decreasing sodium intake by about 0.9 g per day causes a greater reduction in BP when the starting sodium intake is about 2.3 g per day than when it is about 3.5 g per day [14, 15]. However, some researchers believe that the participation of salt in hypertension is far more complex than those reported by previous studies and concluded that the postulate of salt overload leads to long-term negative structural and functional changes of target organs, regardless of its effect on BP [7].

Increased levels of systolic and diastolic BP are associated with the risk for developing coronary heart and cerebrovascular disease. Hypertension is a major risk factor for stroke and is associated with vascular disease in small and large arteries. The risk posed by hypertension is higher for heart failure and stroke, but in the countries of the north west, coronary heart disease is the most common and lethal condition [1, 16]. Therefore, it is natural to think that reducing salt intake, essential part of hypertension treatment, also contributes to the prevention of stroke.

 A meta-analysis of six randomized trials showed that a reduction in dietary salt intake by 2.0–2.3 g (half a teaspoon) per day was associated with a reduction in cardiovascular events by 20% (relative risk 0.80, 95% CI 0.64–0.99) [17]. However, no randomized trials evaluating the effect of salt reduction on risk of stroke or its pathological and etiological subtypes have been done.

Another recent meta-analysis has clearly shown that higher salt intake is associated with a greater incidence of stroke and cardiovascular events. This systematic review identified 13 relevant and suitable studies published from 1996 to 2008. These studies provided evidence from 170,000 people contributing for more than 10,000 vascular events [5].

Many studies have assessed the associations between dietary exposures and cardiovascular or stroke risk. The findings are diverse, mainly because most studies are epidemiological and prone to substantial methodological challenges of bias, confounding factors, and measurement error.

### 2.2. Coffee

Coffee is one of the most frequently consumed beverages worldwide [18]. It has been documented to have acute deleterious physiologic effects within hours after consumption, including increased systolic and diastolic BP, [19] vascular resistance, [20] and impairment of endothelium-dependent vasodilation [21]. Several mechanisms have been proposed, including sympathetic overactivation, increased norepinephrine release, renal effects, and activation on the renin-angiotensin system [19].

On the other hand, compounds in coffee may have beneficial effects on the cardiovascular system. In addition to minerals and trace elements, like potassium, magnesium, and manganese, the coffee is a source of phenolic compounds, including chlorogenic acid. It is possible that the minerals and polyphenols effects on the cardiovascular system would be higher than the adverse effects of caffeine [19]. The phenolic compounds in coffee possess antioxidant capacity and can inhibit the oxidative modification of low-density lipoprotein [22] and may also have effects on serum cholesterol and homocysteine concentrations, oxidation, and inflammation [23].

Chronic coffee consumption has been inconsistently associated with risk of stroke. Lopez-Garcia and colleagues (2009) have found that long-term coffee consumption was not associated with an increased risk of stroke in a prospective cohort of 83,076 women without history of stroke. In contrast, their data suggest that coffee consumption may modestly reduce risk of stroke [24]. Another study, including patients with type 2 diabetes, has observed no association between coffee and risk of total stroke [25].

In a cohort study with male Finnish smokers aged 50 to 69 years without a history of stroke at baseline, Larsson and colleagues [26] showed that coffee consumption was inversely associated with the risk of cerebral infarction, but not intracerebral or subarachnoid hemorrhage, during a mean followup of 13.6 years.

A meta-analysis of 11 prospective studies of 479,689 participants, in which relative risk of stroke for three or more categories of coffee consumption was reported, has concluded that moderate coffee consumption may be weakly inversely associated with risk of stroke [18]. By contrast, in a multicenter case-crossover study with 390 subjects, the subject's acute coffee consumption in the hour before stroke symptoms was compared with his or her usual frequency of consumption in the prior year. The authors have found that coffee consumption transiently increases the risk of ischemic stroke onset, particularly among infrequent drinkers [27].

It is unclear whether the relationship between coffee consumption and stroke is due to potentially unfavorable effects of caffeine or due the polyphenols effects. The studies are controversial and present residual confounding from other stroke risk factors related to coffee consumption. Future studies should attempt to assess this association.

### 2.3. Flavonoids

Flavonoids (flavonols, flavones, and isoflavones) are other dietary antioxidants compounds commonly found in concentrated amounts in multiple fruits, vegetables, and beverages, including apples, berries, grapes, onions, red wine, tea, cocoa, and dark chocolate [28]. They are characterized by their inherent potent antioxidant effects, with a range of biochemical properties, such as antioxidant, antiinflammatory, and antithrombotic effects [29], inhibiting lipid peroxidation, preventing atherosclerosis, promoting vascular relaxation, and with antihypertensive properties [30] that may explain beneficial effects on cardiovascular disease. Hollman and colleagues [29] have conducted a meta-analysis of six prospective cohort studies to assess quantitatively the strength of the association between flavonol intake and stroke incidence. A high intake of flavonols when compared with a low intake was inversely associated with nonfatal and fatal stroke, suggesting that flavonols may reduce stroke risk. During seven years of followup, a cohort study examined the association between flavonoid intake and cardiovascular disease. In this study, flavonoid consumption was associated with lower risk of death from CVD, and total flavonoid intake was more strongly associated with stroke mortality in men. The authors indicated that even relatively small amounts of flavonoid-rich foods may be beneficial [31]. On the other hand, Hirvonen and colleagues have found an inverse association between dietary intake of beta-carotene and the risk for cerebral infarction, but no association was detected between other dietary antioxidants and risk for stroke [32].

Besides flavonoids, some studies have investigated the relationship between foods and beverages that contain polyphenolic compounds, like dark chocolate and tea. The highest levels of chocolate consumption were associated with a reduction in cardiovascular disease and stroke [33, 34]. The same results can be observed when the studies assess the relationship between tea consumption and risk of stroke [35–37].

### 2.4. Carotenoids

Carotenoids, the pigments responsible for the yellow to red color of some fruits and vegetables, have been implicated as beneficial substances; they are found in the human diet and primarily derived from plants and found in roots, leaves, shoots, seeds, fruit, and flowers [38]. Various biological effects have been attributed to carotenoids. A possible mechanism of action is through the antioxidant activity, but other mechanisms of protection may exist [38]. The currently dietetic recommendation to increase consumption of fruits and vegetables rich in antioxidants has generated interest in the role of carotenoids [39], but the mechanisms are not clearly known. Some researchers have evaluated their effects in preventing cardiovascular disease. Prospective studies have shown the association between plasma levels of carotenoids and markers of inflammation, oxidative stress, endothelial dysfunction [40, 41], and arterial stiffness [42]. Their many conjugated double bonds give them an antioxidant potential. Lycopene is the most powerful antioxidant among plasma carotenoids [43]. Its effects have been related to decreased risk of cardiovascular disease [44, 45], including atherosclerosis [46] and myocardial infarction [47]. These nutrients can affect the risk of stroke. The oxidation hypothesis of atherosclerosis points out that oxidation of low-density lipoprotein cholesterol (lipid peroxidation) allows it to accumulate in artery walls promoting atherosclerosis [4].

There are a few studies associating carotenoids with stroke. Rissanen and colleagues, in the Kuopio Ischaemic Heart Disease Risk Factor Study, examined 725 men during six years, associating serum lycopene levels and risk of coronary heart disease and stroke. Men in the lowest quarter of serum lycopene levels had a 3.3-fold risk of acute coronary heart events and stroke. These findings suggest that lycopene might have a role in the prevention of coronary events and stroke [48]. Another study conducted a prospective, nested case-control analysis among male physicians without diagnosed cardiovascular disease followed up for 13 years. Samples from 297 physicians with ischemic stroke were analyzed with paired controls and matched for age and smoking, for 5 major carotenoids (*α*- and *β*-carotene, *α*-cryptoxanthin, lutein, and lycopene), retinol, and *α*- and *γ*-tocopherol, and risk of ischemic stroke was inversely related to plasma levels of carotenoids [49]. Despite these findings, further studies are needed to evaluate the association between carotenoids compounds and the risk of stroke.

### 2.5. Fats

Bioactive compounds of interest in clinical studies due to its antiarrhythmic effect [50], having beneficial effects on multiple cardiac disorders, are represented by n-3-polyunsaturated fatty acids. The major food sources of n-3-polyunsaturated fatty acids are deep and cold water fish such as salmon, trout, and cod. The oils of many species of marine fish are rich in eicosapentaenoic acid (EPA) and docosahexaenoic acid (DHA), the two forms that have long chains and active n-3-polyunsaturated family [51].

Fat is one of the diet variables that exerts direct influence on cardiovascular risk factors [52]. For the treatment of hypercholesterolemia, the dietary recommendations for fat intake must be within a limit of 25–35% of the total calories, divided in ≤7% saturated fats, ≤10% polyunsaturated fats, and ≤20% monounsaturated fats [53]. Many studies suggest that the disproportion in the consumption of n-6 and n-3 fatty acids in the diet can lead to the development of chronic diseases. Before this, global recommendations of n-3 fatty acids for primary prevention of coronary diseases correspond to regular intake of 2 servings of fish per week, equivalent to 1 g of EPA and DHA combination [54].

Although the benefits attributed to fish intake on the cardiovascular mortality have been suggested by observational studies [55–57], the effects of fish consumption on the risk of stroke still remain controversial [58]. There are few studies that have examined the association of different types of fish with the subtype of stroke. According to a population-based prospective study in the Swedish Mammography Cohort, with a mean followup of 10.4 years, women in the highest quintile of fish intake compared to those who never consumed lean fish had a 33% lower risk of total stroke. In multivariate relative risk analysis of total stroke and hemorrhagic stroke, women who consumpted >3 servings of lean fish/week compared to those who consumpted <1 servings of lean fish/week showed relative risk of 0.84 (95% CI: 0.71, 0.98; *P* for trend = 0.049) and 0.67 (95% CI: 0.42, 1.08; *P* for trend = 0.08), respectively [58, 59].

Large clinical trials, such as Diet and Reinfarction Trial (DART) [60] and the Gruppo Italiano per lo Studio della Sopravivenza nell Miocardico Infarction Prevenzione (GISSI), showed clear benefits of n-3-polyunsaturated fatty acids in reducing total mortality and sudden death. On the other hand, the results of controlled clinical trials are inconsistent in relation to the consumption of n-3 and stroke [61].

Observational studies conducted with the alpha-linolenic acid suggest a reduction in 35 to 50% on the stroke risk with increasing its intake [62]. Its main sources are vegetable oils such as soybean, canola, and flaxseed, and also in walnuts [4]. The adoption of the Mediterranean diet, which encompasses the increased alpha-linolenic acid intake, a reduction in saturated fat, and a modest increase in fiber and total carbohydrate, was associated with a 72% reduction in recurrent coronary heart events in patients with prior myocardial infarction [63, 64].

It is well known that the saturated fat influences the plasma lipid levels, particularly in cholesterolemia [53]. Scientific evidence suggests that replacing saturated fat by polyunsaturated or monounsaturated fat is able to reduce both HDL- and LDL-cholesterol. However, the results of dietary interventions involving such a replacement for the reduction of cardiovascular events are not consistent [65].

### 2.6. Carbohydrates

Carbohydrates are the main source of energy for the body and daily nutrient recommendations are based on the Dietary Reference Intakes (DRIs) by age and gender. The Dietary Guidelines for Americans suggest that about half (45–65%) of your daily calories should come from carbohydrates (starches, fiber, and sugars) [66]. However, estimates indicate that the total caloric intake exceeds DRIs regardless of energy needs, and the evidence for the risks imposed on human health by excess carbohydrates consumption might be an increase in the development of insulin resistance, diabetes, stroke, and cardiovascular disease [67–69]. The relationship between intake of sugars and cardiovascular health has emerged since the last American Heart Association (AHA) scientific statement was published in 2002 [70]. In 2006, the AHA published revised diet and lifestyle recommendations that propose minimizing the intake of beverages and foods with added sugars [71].

Strong evidence shows that the total number of calories consumed is the essential dietary factor relevant to body weight gain and adiposity [72], and the quality of carbohydrate intake also affects metabolic health. The proportion of macronutrients takes into account both chronic disease risk reduction and intake of essential nutrients. However, due to the considerable increase in prevalence of obesity and related chronic diseases, the AHA recently released a scientific statement recommending reductions in added-sugar intake to no more than 100 to 150 kcal/d for most Americans [73, 74]. Cross-sectional studies in humans link soft drink consumption with higher energy intake, greater body weight, and poor nutrition [75] and suggest that excessive fructose consumption is playing a role in the epidemics of insulin resistance, obesity, hypertension, stroke, dyslipidemia, and type 2 diabetes mellitus in humans [76–80], whereas overall carbohydrate intake is less strongly related to these diseases.

Currently, increased risk of stroke mortality is associated not only with high intake of carbohydrate, but also with the dietary glycemic index (GI) and glycemic load (GL). A recent study suggests that the risk of mortality from ischemic stroke is increased with increased level of the dietary GI among women [67]. A positive trend is also suggested between dietary GL and mortality from hemorrhagic stroke among women [81, 82]. A meta-analysis study provides high-level evidence that diets with a high GI, high GL, or both, independently of known confounders, including fiber intake, increase the risk of chronic lifestyle-related diseases. Overall, GI had a more powerful effect than did the GL, with more positive associations between GI and chronic disease risk [83, 84].

It is known that the increased fiber intake reduces blood pressure, blood glucose, serum triglycerides, and LDL cholesterol [4, 85] and may decrease the risk of chronic diseases by improving the postprandial glycemic response and insulin concentration. The type of fiber could be differentially related to risk of the diseases. Instead, recent study suggested that the risk of stroke is decreased with increased whole grain intake and was associated with micronutrients that accompany fiber in whole-grain cereal, contributing to lower risk of stroke [82]. Some studies suggest that whole grain or cereal fiber intakes as a source of fiber, among different sources of fiber, were inversely associated with risk of stroke in US men [82, 85].

## 3. Conclusion

In summary, evidence shows that association between the magnitude of salt intake and hypertension may increase risk in both myocardial infarction and stroke. Moreover, the excessive intake of nutrient-dense forms of foods has become compelling, and the population has difficulty in following low-calorie diet to promote weight loss over time. Sugar-sweetened beverages are contributors to add sugar intake and weight gain and can lead to increased risk of stroke. A diet rich in whole grains, fruits, and vegetables may help reduce body weight and provide adequate amounts of flavonoids, carotenoids, minerals, and trace elements, which can help to reduce chronic disease risk and stroke.
